# A lack of commensal microbiota influences the male reproductive tract intergenerationally in mice

**DOI:** 10.1530/REP-24-0204

**Published:** 2025-03-04

**Authors:** Natalie A Trigg, Simon K Zhou, Jordan C Harris, Madeline N Lamonica, Molly A Nelson, Michael A Silverman, Taku Kambayashi, Colin C Conine

**Affiliations:** ^1^ Division of Neonatology, The Children’s Hospital of Philadelphia, Philadelphia, Pennsylvania, USA; ^2^ Departments of Genetics and Pediatrics – Penn Epigenetics Institute, Institute of Regenerative Medicine, and Center for Reproduction and Women’s Health, University of Pennsylvania Perelman School of Medicine. Philadelphia, Pennsylvania, USA; ^3^ Department of Pathology and Laboratory Medicine, Perelman School of Medicine at the University of Pennsylvania, Philadelphia, Pennsylvania, USA; ^4^ Department of Dermatology, Perelman School of Medicine at the University of Pennsylvania, Philadelphia, Pennsylvania, USA; ^5^ Division of Infectious Disease, Department of Pediatrics, The Children’s Hospital of Philadelphia. Philadelphia, Pennsylvania, USA; ^6^ Institute for Immunology and Immune Health and Department of Microbiology, Perelman School of Medicine, University of Pennsylvania, Philadelphia, Pennsylvania, USA; ^7^ Department of Microbiology, Perelman School of Medicine, University of Pennsylvania, Philapdelphia, Pennsylvania, USA

**Keywords:** epididymis, microbiome, spermatozoa, testis, non-genetic inheritance, T cells

## Abstract

**In brief:**

Germ-free mice display epididymal transcriptomic changes that were also evident in their conventionalized male offspring and mice lacking T and B cells. This paper demonstrates the role of microbiota and immune cells in the epididymis.

**Abstract:**

The microbiome encompasses the array of microorganisms inhabiting various niches in the body and is necessary for numerous physiological processes, including normal metabolism and a functioning immune system. Not only does the absence of a microbiome in mice impact the exposed animals but also inherited phenotypes in successive generations of progeny, suggesting that the absence of a microbiome impacts the germline and gametes. Indeed, recent research has identified a role of the gut microbiome in contributing to male fertility, in both healthy and disease states. While this link is beginning to be established, the impact of the microbiome on the male reproductive tract remains understudied. Here, we utilized a germ-free mouse model to examine the influence of the absence of microbes on the male reproductive tract. In contrast to mice with an established microbiome, germ-free mice display decreased testicular weight and the prevalence of an epididymitis-like inflammation phenotype. These histopathological changes are accompanied by transcriptomic dysregulation in the reproductive tract of germ-free mice, particularly in the cauda epididymis. Moreover, these transcriptomic changes are transmitted to the next generation with high correlation of gene expression in the cauda epididymis between germ-free mice and their conventionalized (microbiome-restored) male offspring, when compared to control mice. Ultimately, our findings identify the reproductive sequalae of males without a functional microbiome and additionally in their conventionalized offspring, suggesting that the paternal microbiota is an underappreciated contributor to male reproductive function.

## Introduction

The vast and symbiotic collection of bacteria, viruses, fungi and archaea that inhabit different niches in the body, such as the gastrointestinal tract, skin and respiratory tract, are collectively known as the microbiome (Hou *et al.* 2022). Since its discovery, the role of the microbiome in human health and disease has been widely reported. Indeed, the human gut microbiome is readily involved in nutrient extraction, metabolism and immunity. In addition, disruption of the balance of microbes has been associated with numerous chronic diseases including diabetes, cardiovascular disease and gastrointestinal syndromes such as irritable bowel syndrome (Ponnusamy *et al.* 2011, Kostic *et al.* 2015, Tang *et al.* 2017, Hou *et al.* 2022, Vijay & Valdes 2022). Similarly, dysbiosis in the skin can cause acne and atopic dermatitis (Ta *et al.* 2020). These insights into the contribution of the microbiome to health and the mechanisms involved have largely been characterized through the use of axenic or germ-free (GF) mouse models (Kennedy *et al.* 2018). These mice are reared under sterile conditions and therefore, have not been naturally colonized by microorganisms are typically compared to specific pathogen-free (SPF) mice, which possess a normal commensal microbiome but are free of pathogenic strains. The use of these GF mouse models has identified the requirement of the microbiome for the normal function of many systems, including the immune and nervous systems and metabolism (Luczynski *et al.* 2016, Kennedy *et al.* 2018). In line with this, recent research from our laboratory using GF mice showed that microbes influence sebaceous gland function and modify the transcriptional profile of multiple organs, including the liver and small intestine (Harris *et al.* 2024). Surprisingly, these phenotypes were also present in succeeding generations of progeny with a normal functioning microbiome, indicating the transmission of epigenetically inherited phenotypes from a GF parent to offspring. Hence, this finding suggests that the absence of microbiota could influence the reproductive tract of GF mice to alter the epigenetic information in GF gametes and influence the next generation.

There have been several previous studies that report the identification and role of immune cells within the male reproductive tract (Guiton *et al.* 2019, Bhushan *et al.* 2020, Pleuger *et al.* 2022). Indeed, within the reproductive organs, immune cells are crucial to maintaining homeostasis and supporting normal physiological processes. These functions are of particular importance, given that the production of sperm occurs after self-immunity has been established; therefore, the male reproductive tract requires a balance of maintaining an immunotolerant environment to sperm cells, while providing protection from pathogens (Voisin *et al.* 2019). Such importance is evidenced by the finding that 15% of male infertility cases are attributable to immunological disorders (Guiton *et al.* 2019). As it is well-established that the presence and composition of the microbiome influences the function of the immune system and owing to the dynamic relationship between reproduction and somatic health, it is no surprise that the microbiome additionally influences male fertility (Salonia *et al.* 2009, Burke *et al.* 2022). Indeed, studies have uncovered associated changes in sex hormones, sperm production and testicular function correlated with alterations in the gut microbiome in humans (Shin *et al.* 2019), with similar findings also recapitulated in mouse models (Al-Asmakh *et al.* 2014). Furthermore, several disease pathologies that display associated male infertility, such as high-fat diet and type-1 diabetes, equally produce alterations in the microbiome, with paternal exposure also leading to transmitted phenotypes in offspring, including reduced fertility (Bakos *et al.* 2011, Shayeb *et al.* 2011, Muscogiuri *et al.* 2019, Liu *et al.* 2021). While many mechanisms could conceivably lead to the induced infertility, such as metabolic syndrome, sperm DNA damage or altered hormone levels, a recent study has examined the mechanistic link between high-fat diet-induced infertility and gut dysbiosis. Through restoration of the gut microbiome via fecal microbiome transplantation, semen quality in high-fat diet-fed mice was improved. Specifically, fecal microbiome transplantation led to increased sperm concentration and motility and improved pregnancy rates compared to high-fat diet-fed mice that did not receive the transplantation (Hao *et al.* 2022*a*). Likewise, this approach was also sufficient in rescuing sperm parameters in a mouse model of type-1 diabetes (Hao *et al.* 2022*b*). Albeit, whether other disease parameters were also improved following fecal microbiome transplantation was not explored, and therefore, whether the restoration of the gut microbiome or subsequent amended disease state led to improved fertility parameters remains to be determined. Nevertheless, these findings highlight the contribution of the microbiome to reproductive health and may signify a clinical target for male infertility.

The prevalence of male infertility is growing globally. Remarkably, the underlying cause of the vast majority of infertility cases in men remains unknown (Kimmins *et al.* 2024). While assisted reproductive technologies (ART) are available to bypass these issues, the inability to define the underlying cause of male infertility places the burden of this issue on the woman through forced adoption of ART. This in turn also impedes the ability to diagnose comorbidities in the male, which are common with an infertility diagnosis (Shiraishi & Matsuyama 2018, Burke *et al.* 2022). Moreover, mouse models of paternal environmental exposures, such as high-fat diet, result in subfertility in the exposed animals. In addition, these exposed males sire offspring with altered phenotypes, such as metabolic phenotypes, suggesting a phenomenon in which fertility issues in the exposed generation may proceed the transmission of phenotypes to the next generation (Fullston *et al.* 2013, Fullston *et al.* 2015, Rahman *et al.* 2020, Zatecka *et al.* 2021). Supportive evidence of this also occurring in humans highlights the potential health burden that can be placed on the next generation when causes of infertility are overlooked and highlights the importance of addressing infertility, not only to achieve fertilization but also for the long-term health of successive generations of progeny (Sansone *et al.* 2018, Dimofski *et al.* 2021, Jawaid *et al.* 2021, Montagnoli *et al.* 2021). While it is becoming established that disruption in the microbiome influences male fertility parameters, the impact on the male reproductive tract remains largely unknown but represents a potential avenue that contributes to declining fertility and the transmission of non-genetically inherited offspring phenotypes. Hence, here we utilized GF mice to examine how the absence of the microbiome influences the male reproductive tract histology and gene expression and determine the impact of a GF father on the reproductive tract of male offspring.

## Materials and methods

### Ethics

All animal work was regulated according to ethical review by the University of Pennsylvania Institutional Animal Care and Use Committee, IACUC# 804245. All mice were housed in temperature- and humidity-controlled conditions under a 12h light:12h darkness cycle and fed a standard chow diet *ad libitum*.

### Animals and breeding

All SPF mice were derived from C57BL/6 mice purchased from Charles River Laboratories (strain number 556) and used as controls in this study. GF mice were acquired from the University of Pennsylvania Gnotobiotic Core, which houses C57BL/6 colonies in sterile isolators. SPF and GF breeding pairs were established in a conventional mouse facility at the University of Pennsylvania to produce F1 progeny that are assessed in these studies. Recolonized GF mice were obtained from the Silverman Laboratory at the Children's Hospital of Philadelphia (CHOP).

### Reproductive organ assessment and sperm parameters

Adult male mice (8–14 weeks) were euthanized, weighed and dissected for the male reproductive tract. Testes and epididymides were dissected and cleaned of any residual fat and weighed. For histology, the testis and epididymis were immersed in 10% buffered formalin and fixed for 48 h before washing (twice) and storing in 50% ethanol. Fixed tissue was paraffin-embedded using standard procedures and 5 μm sections were cut using a microtome. Hematoxylin and eosin (H&E) staining was performed for histopathology assessment by the University of Pennsylvania, School of Veterinary Medicine Comparative Pathology Core. Tissue sections were visualized and imaged on a Aperio VERSA 200 platform (Leica Microsystems, Germany). The epididymis was further divided into caput (proximal) and cauda (distal) segments and tissue was washed in PBS and snap frozen in liquid nitrogen, ready for RNA extraction.

Sperm was retrieved from the cauda epididymis by immersing the dissected tissue in 1.5 mL Biggers, Whitten and Whittingham (BWW) media (91.5 mM NaCl, 4.6 mM KCl, 1.7 mM CaCl_2_·2H_2_O, 1.2 mM KH_2_PO_4_, 1.2 mM MgSO_4_·7H_2_O, 25 mM NaHCO_3_, 5.6 mM d-glucose, 0.27 mM sodium pyruvate, 44 mM sodium lactate, 5 U/mL penicillin, 5 μg/mL streptomycin, 20 mM HEPES buffer and 1 mg/mL polyvinyl alcohol) and with blunt forceps, gently squeezing luminal contents into the media. Following a swim-out incubation of 10 min at 37 °C, the sperm suspension was transferred to a 1.5 mL tube and allowed to swim up for 5 min at 37 °C. At this time, an aliquot of sperm solution was taken to assess sperm motility and vitality. Both parameters were assessed using phase microscopy and at least 100 cells per sample were recorded. Vitality was evaluated using trypan blue vitality stain, with dye exclusion indicating an intact membrane and hence, vitality.

### Immunofluorescence of epididymal sections

Immunofluorescence staining of epididymal sections was performed at the University of Pennsylvania, School of Veterinary Medicine Comparative Pathology Core, as previously detailed (Radaelli *et al.* 2023). A Leica BOND RXm automated platform combined with the OPAL Automation Multiplex IHC Detection Kit (Akoya Biosciences, USA, NEL830001KT) implemented onto a Leica BOND Research Detection System (DS9455) was used. Briefly, tissue sections were deparaffinized and rehydrated using standard procedures and pretreated with the epitope retrieval BOND ER2 high pH buffer (Leica AR9640) for 20 min at 98 °C. All subsequent incubations were performed at room temperature. Endogenous peroxidase was inactivated with 3% H_2_O_2_ for 10 min and sections were blocked for 30 min with the Akoya Biosciences Opal Antibody Diluent/Block solution (ARD1001EA). Sections were then incubated in primary antibodies against F4/80 (1:500, Cell Signaling Technology, USA, 70076) for 45 min and appropriate secondary antibodies for 25 min. Finally, sections were incubated in the Akoya Biosciences TSA reagents Opal 520 (OP-1001) for 10 min, followed by Spectral DAPI nuclear counterstain (Akoya Biosciences FP1490) and mounted with Fluoromount-G (SouthernBiotech, USA 100-01). Immunofluorescence signal was captured using a Aperio VERSA 200 (Lecia). Negative controls were obtained by replacement of the primary antibodies with irrelevant isotype-matched anti-rabbit antibodies. The ImageJ software (NIH, USA) was used to quantify fluorescent signal and is reported as corrected total cell fluorescence (Ansari *et al.* 2013).

### *In vitro* fertilization (IVF)

Cauda epididymides from SPF and GF male mice were dissected and sperm was retrieved by retrograde perfusion and capacitated for 45 min in BWW media supplemented with 1.0 mg/mL methyl-β-cyclodextrin at 37 °C in an atmosphere of 5% CO_2_ and 5% O_2_. Eggs were retrieved from superovulated (5IU PMSG, followed by 5IU hCG 48 h later) SPF female mice 15 h after hCG injection. Cumulus masses were washed in high-calcium human tubal fluid (HTF) and transferred to a droplet of HTF supplemented with 1.0 mM reduced glutathione ready for IVF, as previously described (Trigg *et al.* 2021). Eggs from SPF female mice were split into two groups, one to be fertilized by SPF sperm and the other, GF sperm. Two × 10^5^ sperm were deposited into the egg containing droplet and allowed to coincubate for 3 h at 37 °C, 5% CO_2_ and 5% O_2_. After coincubation, presumptive zygotes were retrieved and washed in KSOM and cultured for 24 h. Fertilization rate was determined by recording the number of two-cell embryos at 24 h and reporting as a percentage of eggs in the fertilization droplet.

### RNA extraction

Frozen tissue (*n* = 3–6 biological replicate per tissue, with an individual biological replicate consisting of tissue from one animal) was thawed on ice and transferred to screw top tubes with equal volume of 0.1 mm silicone beads. 200 μL Trizol was added and tissue was homogenized at 2,000 rpm for 2 min. Lysed tissue was transferred to a phase lock tube (Quantabio, USA, 2302830) with 0.2 × volume of BCP (1-bromo-2 chloropropane) and centrifuged at 14,000 ***g*** for 4 min at 4 °C. The aqueous layer was transferred to a fresh tube and 20 μg glycoblue (Thermo Fisher, USA; AM9516) and 1.1 × volume of isopropanol was added. RNA was precipitated for at least 1 h at −20 °C before cold 70% ethanol wash and reconstitution in nuclease-free water. Genomic DNA was removed by treatment with DNase I (QIAGEN, 79254) for 15 min at room temperature, as per manufacturer’s instructions. RNA was quantified using the Nanodrop 2000 (ThermoFisher, ND-2000) and aliquoted ready for RNA-sequencing.

### RNA-sequencing

For each sample, 1 μg of RNA was used to generate RNA-seq libraries using the Illumina Stranded mRNA kit. Briefly, oligo(dT) magnetic beads were used to capture messenger RNAs from the testis and epididymal tissue total RNA. The selected RNA was then fragmented and primed for cDNA synthesis, as per the manufacturer’s instructions. A second strand synthesis was performed with dUTP replacing dTTP to achieve strand specificity. An adenine and a thymine nucleotide were added to the 3′ end to allow for ligation. The resulting products were cleaned up using AMPure XP beads (Beckman Coulter, USA, A63881) and amplified and combined at equimolar concentrations before purification using non-denaturing PAGE. Libraries were loaded onto an Illumina NextSeq 1000 and sequenced using paired-end technology (50 bp per read). Data were mapped using RSEM against the *Mus musculus* genome (mm10) and normalized to transcript length and library size (transcript per million, TPM) using the Via Foundry platform (Yukselen *et al.* 2020). To assess the differential abundance of transcripts between SPF and GF tissues, raw count data were imported into the R Statistical software (https://www.r-project.org/) and analyzed using the DESeq2 package (Love *et al.* 2014). Differentially abundant genes were determined as those with a fold-change ≥1.5 and false discovery rate (FDR) adjusted *P*-value (*P*-adj) ≤ 0.05.

### *In silico* analysis

RNA-sequencing datasets were uploaded to the Ingenuity® Pathway Analysis (IPA) software (QIAGEN, Germany) to assess the enrichment of cellular location and molecular function of the list of differentially expressed genes (DEGs) and determine the functional pathways altered in the reproductive tissue of GF mice. To compare DEGs between two groups, Venny 2.0.2 was used (publicly available at http://bioinfogp.cnb.csic.es/tools/venny/index.html). To interrogate our RNA-seq datasets for the presence of immune cell markers, we utilized the publicly available software, ImmunCellAI http://bioinfo.life.hust.edu.cn/web/ImmuCellAI/; (Miao *et al.* 2021).

### Statistical analysis

Data presented in this manuscript are expressed as the mean values ± standard deviation (SD). Statistical analyses were performed using the GraphPad Prism software (https://www.graphpad.com/features; v 10.0.3), using unpaired Student’s *t* test to determine statistical significance. *P*-value <0.05 was considered significant, with the level of significance denoted above the graph by asterisks such that *P* < 0.05 (*), *P* < 0.01 (**), *P* < 0.001 (***) and *P* < 0.0001 (****).

## Results

### The absence of microbes from birth alters adult testis weight and induced cell infiltration in the cauda epididymis

**Figure 1:**
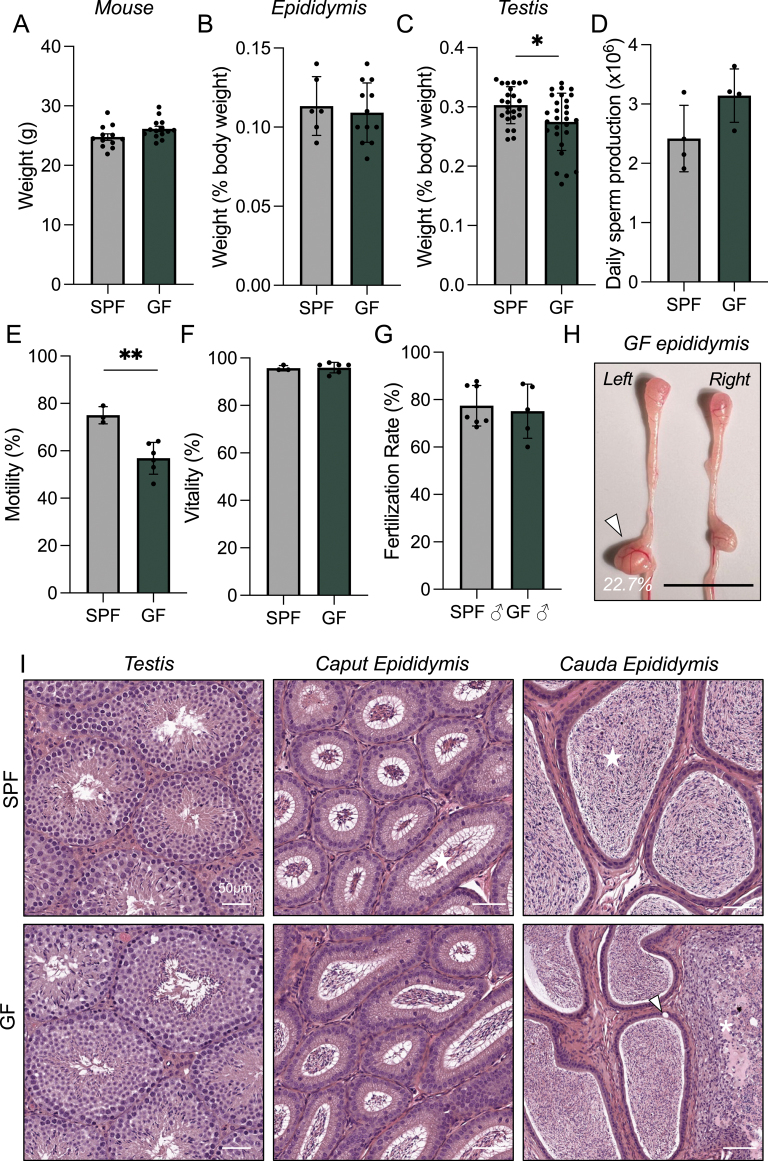
The absence of microbiota impacts the male reproductive tract. (A) SPF and GF adult male mice were euthanized and weighed. The (B) epididymis and (C) testis of each mouse was weighed and is reported as a percentage of mouse body weight. (D) Daily sperm production was calculated on testes collected from SPF and GF mice at 8 weeks of age. Populations of isolated cauda sperm were assessed for (E) motility and (F) vitality using phase microscopy. At least 100 cells were recorded and represented as a percentage. (G) Spermatozoa from SPF or GF males were capacitated and incubated with eggs from SPF females to assess IVF rates. Fertilization rate is presented as a percentage of two-cell embryos over the number of eggs in the fertilization dish. (H) Representative image of epididymides isolated from a GF animal depicting an abnormally large and swollen cauda epididymis (indicated with white arrow). Scale bar = 1 cm. (I) Testis and epididymis histology was assessed in tissue sections stained with hematoxylin and eosin. Scale bar = 50 μm. Sperm build up and cell infiltration in GF cauda epididymis is indicated with an asterisk. White arrow indicates vacuole in epididymal epithelium. All graphical data is plotted as the mean ± SD. Differences between groups were assessed with (A, B, D, E, F, G) unpaired Student’s *t*-test or (C) nonparametric Mann–Whitney test. Asterisks (*) indicates *P* < 0.05 and **P < 0.01.

SPF (also referred to as control mice) and GF adult male mice (8–10 weeks old) were euthanized and dissected for the male reproductive tract. The absence of microbiota did not influence mouse weight or the weight of the epididymis (Fig. 1A and B). Of note, however, we did detect a significant decrease in testicular weight in GF males (Fig. 1C). However, this did not translate to a change in daily sperm production in GF mice (Fig. 1D). Subsequent investigation into sperm parameters revealed a significant decrease in total sperm motility (18% reduction) compared to sperm from SPF males (Fig. 1E). The attenuated motility was not accompanied by a reduction in sperm vitality, as the percentage of viable sperm was comparable between SPF and GF males (Fig. 1F). Despite the observed reduced motility of GF sperm, there was no concomitant impact on fertilization rate assessed via IVF. In fact, GF sperm achieved similar fertilization rates to populations of SPF sperm (Fig. 1G).

**Figure 2:**
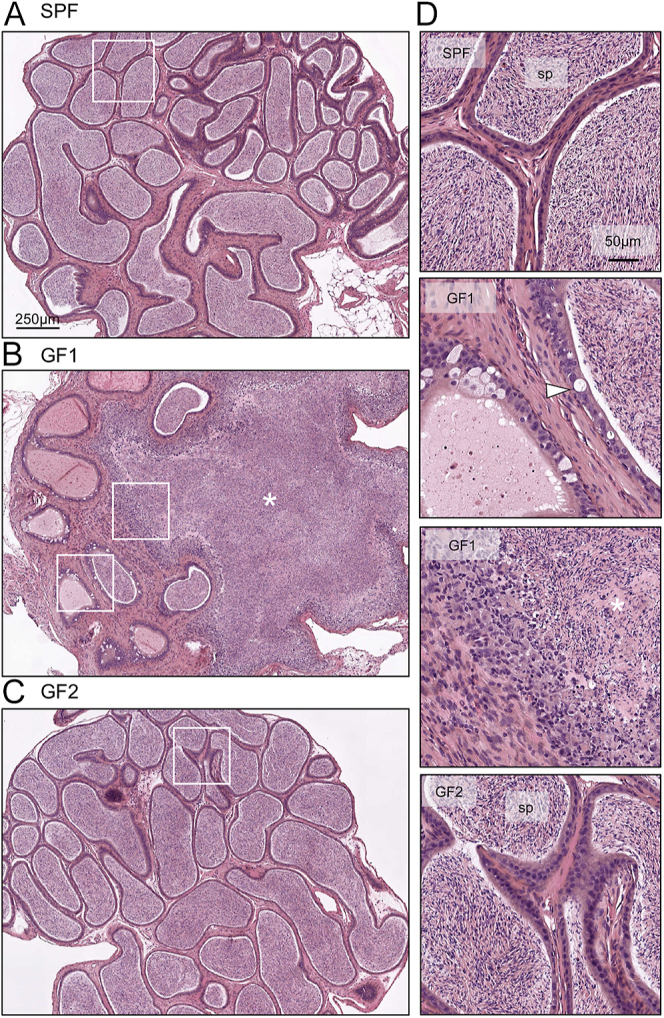
Histological assessment of SPF and GF cauda epididymis. The cauda epididymis from (A) SPF and (B, C) GF male, with (B) swollen phenotype and (C) normal structure. Scale bar = 250 μm and white boxes indicate location of higher magnification images in (D). (D) Higher magnification images of SPF and GF cauda epididymis. Scale bar = 50 μm. White arrow indicates vacuole in epithelium and asterisks indicate sites of cell infiltration in the interstitium. sp = spermatozoa.

While we noted no difference in epididymal weight across the 12 males assayed, we did notice a ‘swollen’ phenotype in some of the epididymides of GF mice. Indeed, of all GF mice euthanized, 22.7% of the males (ten of 44 GF males scarified over the period of this study) displayed the observed ‘swollen’ epididymis phenotype (Fig. 1H). Most commonly, this ‘swollen’ pathology was observed in the cauda epididymis but did not always occur in both epididymides from the same mouse and was not seen in any SPF males (Fig. 1H). The detection of this pathology prompted subsequent histological analysis of GF reproductive tissues. For this, we examined four SPF and GF testes and epididymides (Fig. 1I). Examination of GF testis sections revealed no overt morphological abnormalities when compared to SPF testis (Fig. 1I). The epididymis sections from SPF males displayed a normal histological structure, with the caput and cauda epididymal lumen filled with spermatozoa (Figs 1I and 2A; star). Similarly, the caput epididymis of GF males was filled with sperm and displayed a normal histological structure. Conversely, the cauda epididymis of GF males displayed significant cell infiltration, reminiscent of epididymitis (Figs 1I and 2B, (Liu *et al.* 2020)). Vacuoles were noted in the epithelial cell layer of GF cauda epididymis (Figs 1I and 2D, white arrow), indicating the compromise of the epithelial cell barrier. This was further evidenced by the reduction in tubules and the presence of spermatozoa in the interstitium (Fig. 2B and D; asterisks). Moreover, a portion of the intact tubules of GF cauda were devoid of spermatozoa (Fig. 2B). The degree of severity of the tubule breakdown and cell infiltration varied between GF cauda epididymides, with cell infiltration encompassing ∼80% of the cauda in some cases (Fig. 2B) and 50% in others (Fig. S1A (see section on Supplementary materials given at the end of the article)), while some GF cauda epididymides also appeared indistinguishable from SPF cauda (Fig. 2C).

### GF fathers sire male offspring with altered reproductive tracts

**Figure 3:**
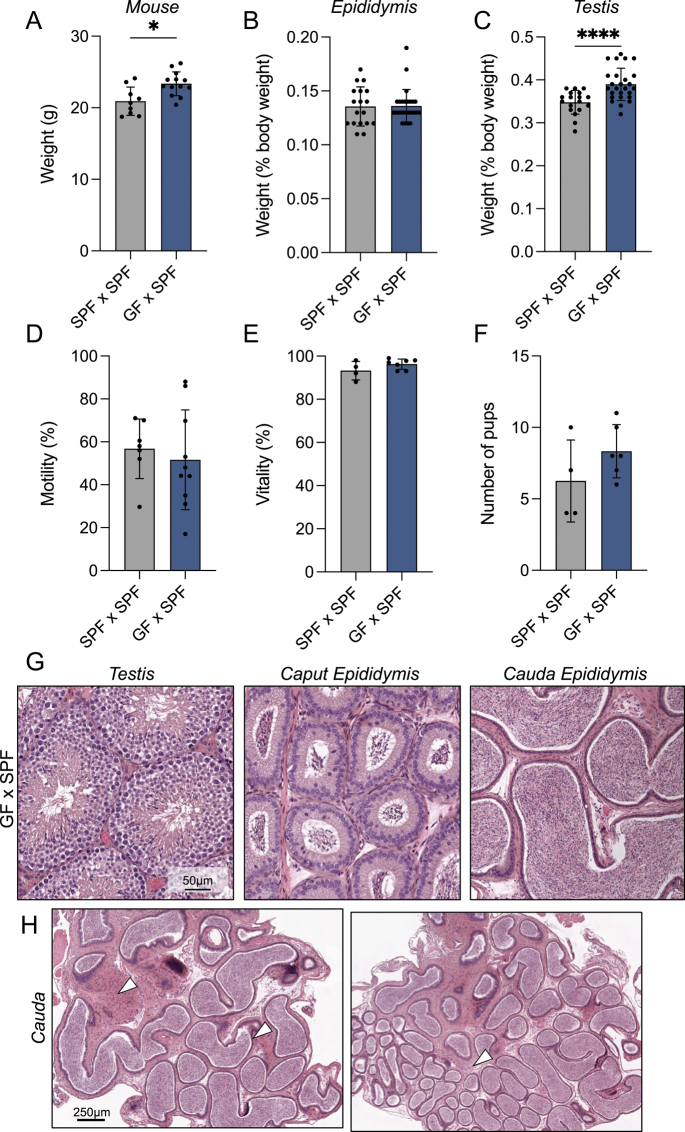
F1 offspring of GF fathers exhibited increased testicular weight and epididymal cell infiltration. (A) Mouse weight, (B) epididymal weight and (C) testis weight of F1 male offspring of SPF × SPF and GF × SPF breeding pairs. Organ weights are depicted as a percentage of mouse body weight. Cauda spermatozoa from male offspring were isolated and (D) sperm motility and (E) vitality was recorded. These measurements were determined by recording at least 100 cells for each sample using phase microscopy. (F) The number of pups born for each litter was recorded and presented for each breeding pair. For A, B, C, D, E, F, each dot indicates a measurement from a single mouse, testis or epididymis. (G) Histological sections of testis and epididymis from GF × SPF male offspring. Scale bar = 50 μm. (H) Histological images of entire cauda epididymis from two GF × SPF male offspring. Scale bar = 250 μm. White arrow indicates potential cell infiltration in interstitium. Graphical data is represented as the mean ± SD. Differences between groups was assessed with one-way ANOVA with Tukey’s multiple comparison test. * indicates *P* < 0.05 and ****P < 0.0001.

We previously showed that GF mice bred with SPF mice transmit multiple phenotypes from the GF parents to offspring, including a sebum secretion defect and transcriptomic changes in the liver and sebaceous glands (Harris *et al.* 2024). Thus, we next sought to determine whether offspring of a GF father mated with a SPF mother displayed any reproductive phenotypes. For this, GF mice were delivered to our conventional animal facility and mated with SPF females to generate offspring. Their progeny was then compared to age-matched offspring derived from SPF parents. It should be noted that as the breeding occurred in a conventional animal facility, any differences seen in F1 offspring is a direct result of having a GF father, as the offspring are exposed to microbes, as an SPF animal would from birth, and display similar skin and gut microbiota to control animals (Harris *et al.* 2024). At adulthood (>8 weeks of age), F1 male offspring were euthanized and dissected for the male reproductive tract. There was a significant increase in body weight of GF × SPF F1 males compared to SPF × SPF controls (Fig. 3A). Epididymal weight was consistent across both groups examined, while testicular weight was significantly increased in GF × SPF offspring (Fig. 3B and C). Interestingly, the 10% increase in testicular weight of GF × SPF F1 mice is in stark contrast to the testicular phenotype (decreased mass) in F0 GF males (Fig. 1C). Sperm parameter assessment revealed no significant difference in motility or vitality between SPF offspring or male offspring derived from GF fathers (Fig. 3D and E). In contrast to F0 GF males, we did not observe the ‘swollen’ epididymis phenotype in F1 offspring while dissecting. Nevertheless, we performed histological analysis on these mice, which revealed no gross abnormalities in the testis or epididymis sections from GF × SPF F1 males. However, there was evidence of potential cell infiltration in the interstitium (Fig. 3G and H, white arrow) not seen in SPF males or their progeny.

### Transcriptomic changes in the testis and epididymis of GF mice

**Figure 4:**
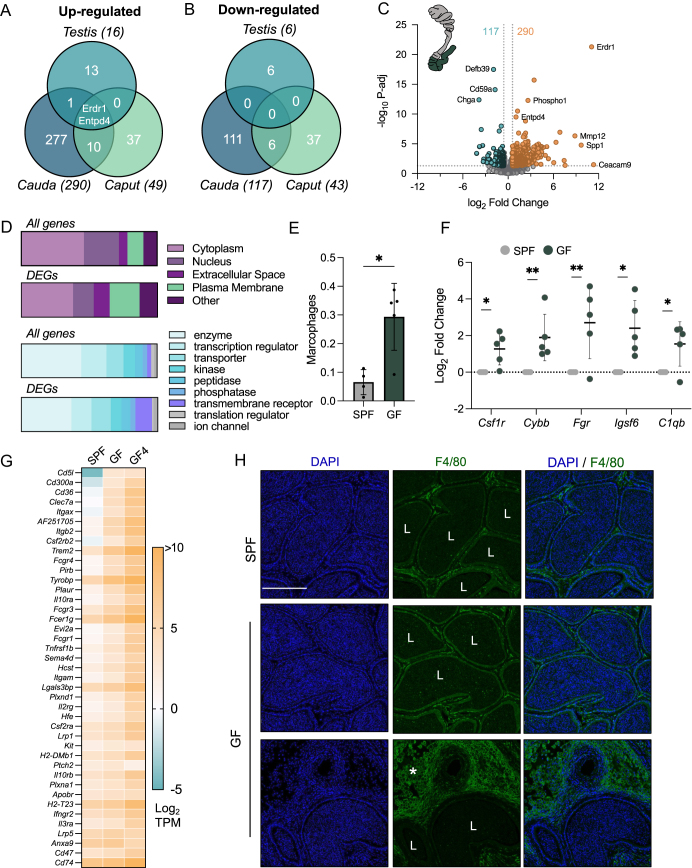
Significant transcriptomic changes occur in the GF cauda associated with immune cells. (A, B) Venn diagrams depict the overlap of DEGs that are (A) upregulated and (B) downregulated in GF reproductive tissues compared to SPF mice. (C) Volcano plot illustrating the log_2_ fold change (*x*-axis) and log_10_ adjusted *P*-value (P-adj; *y*-axis) of genes detected in the GF cauda compared to the SPF cauda. Dots highlighted orange and green indicate those genes that satisfy the criteria of fold change >±1.5 and *P*-adj ≤0.05 and therefore identified as significantly altered. (D) Bar plot indicating the distribution of gene location (top) and type (bottom) in all genes and DEGs in the GF cauda epididymis as determined by Ingenuity Pathway Analysis. (E, F) Expression data were input into the ImmunCellAI to infer the differences in immune cell populations between SPF and GF tissue based on gene expression of cell markers. (E) Abundance score for macrophages in SPF and GF cauda tissue and (F) Log_2_ fold change of five macrophage cell markers in the GF cauda epididymis when compared to SPF as measured by RNA-seq. (G) Heatmap depicting the log_2_ average TPM of SPF and GF cauda and log_2_ TPM for replicate 4 of the GF cauda (GF4) from RNA-seq for 41 genes increased in the GF cauda epididymis and classified as transmembrane receptor. (H) Immunofluorescence staining of cauda epididymis with antibodies against the macrophage marker F4/80 (green) and co-stained with DAPI (blue) from SPF and GF mice. Scale bar = 200 μm. L – lumen and asterisks indicate infiltration of macrophages in the interstitium.

Next, we sought to examine the transcriptomic signature underlying the reproductive phenotype of F0 GF mice and determine the legacy of this alteration in the next generation. Hence, we collected testis, proximal (caput) and distal (cauda) epididymal tissue, isolated total RNA and cloned mRNA-seq libraries (Fig. S2A, Table S1). Notably, we recorded consistent results across biological replicates for each tissue type we analyzed as reported by the principal component analysis and Pearson correlation analysis (Fig. S2B and C). One biological replicate of GF cauda epididymis was identified as an outlier compared to other GF cauda samples (Fig. S2C). This replicate was a ‘swollen’ cauda epididymis and due to the large amount of cell infiltration in these epididymides (Fig. 2B), we removed this sample from our analysis and only referred to it as a separate entity. In assessing the DEGs between SPF and GF reproductive tissues, it was determined that the cauda epididymis displayed the greatest number of altered genes in GF mice compared to SPF mice (Fig. 4A and B). Testis tissue from GF mice expressed 22 genes with altered levels compared to SPF testis, most of which were upregulated (16 genes), while the remaining six genes were downregulated (Fig. S2D). In the caput epididymis, 43 genes were downregulated and 49 genes were upregulated in GF compared to SPF (Fig. S2E). Comparatively, the cauda epididymis displayed the greatest changes in the transcriptome, with 117 genes downregulated and 290 upregulated when GF cauda was compared to SPF (Fig. 4C). Genes of interest included *Erdr1*, which along with *Entpd4*, was increased in expression in all GF tissues analyzed (Fig. 4A).

Using the Ingenuity Pathway Analysis (IPA) software, we interrogated the genes significantly altered in the GF cauda epididymis and revealed an enrichment in genes located in the plasma membrane and classified as ‘transmembrane receptors’ (Fig. 4D, Table S2). Indeed, 43 genes were sorted into this category and of note, 41 of them were significantly increased in expression in the GF cauda compared to the SPF cauda (Fig. 4G). Interestingly, the expression of several of these genes in the excluded (swollen) GF cauda sample (GF4) was even greater than the GF cauda average, suggesting the likely presence of these genes in the infiltrated cells. Some of these genes are immune-related, including *Cd74*, *Itgam* and *Tyrobp* (Leng *et al.* 2003, Liang *et al.* 2021). Accordingly, assessment of the list of DEGs in the GF cauda epididymis identified enriched disease functions of inflammatory response and immunological diseases (Fig. S4A), immune activation pathways, such as neutrophil degranulation and cytokine signaling, and inhibition of cell cycle checkpoints and DNA replication (Fig. S2F, Table S3). The identification of these immune-related pathways and increased receptor expression prompted examination of known immune cell markers in GF tissue. For this, we entered our expression data into an online tool, ImmunCellAI, which generates an abundance score for 24 different immune cell populations in the input data based on the expression level of multiple cell markers (Miao *et al.* 2021). This analysis revealed a predicted increased abundance of macrophages in the GF cauda compared to the SPF cauda (Fig. 4E and S4B). Consistent with this result was the significant increase in five genes in the GF cauda that are known markers of macrophages (Fig. 4F). To confirm this finding, we performed immunofluorescence analysis using antibodies against F4/80, a protein marker of macrophages, within the GF epididymis (Fig. 4H, S4F, G). F4/80 localized to sites of cell infiltration and cells within the interstitium in the cauda epididymis of GF mice (asterisks). Quantitative analysis of F4/80 fluorescence signal across the cauda epididymis revealed a significant increase in F4/80 fluorescence in the GF cauda compared to the SPF cauda (Fig. S4C). F4/80 signal predominantly occurred surrounding sperm cells that had escaped into the interstitium (Fig. 4H, asterisks).

### Epididymal transcriptomic changes associated with GF mice persist to the next generation

**Figure 5:**
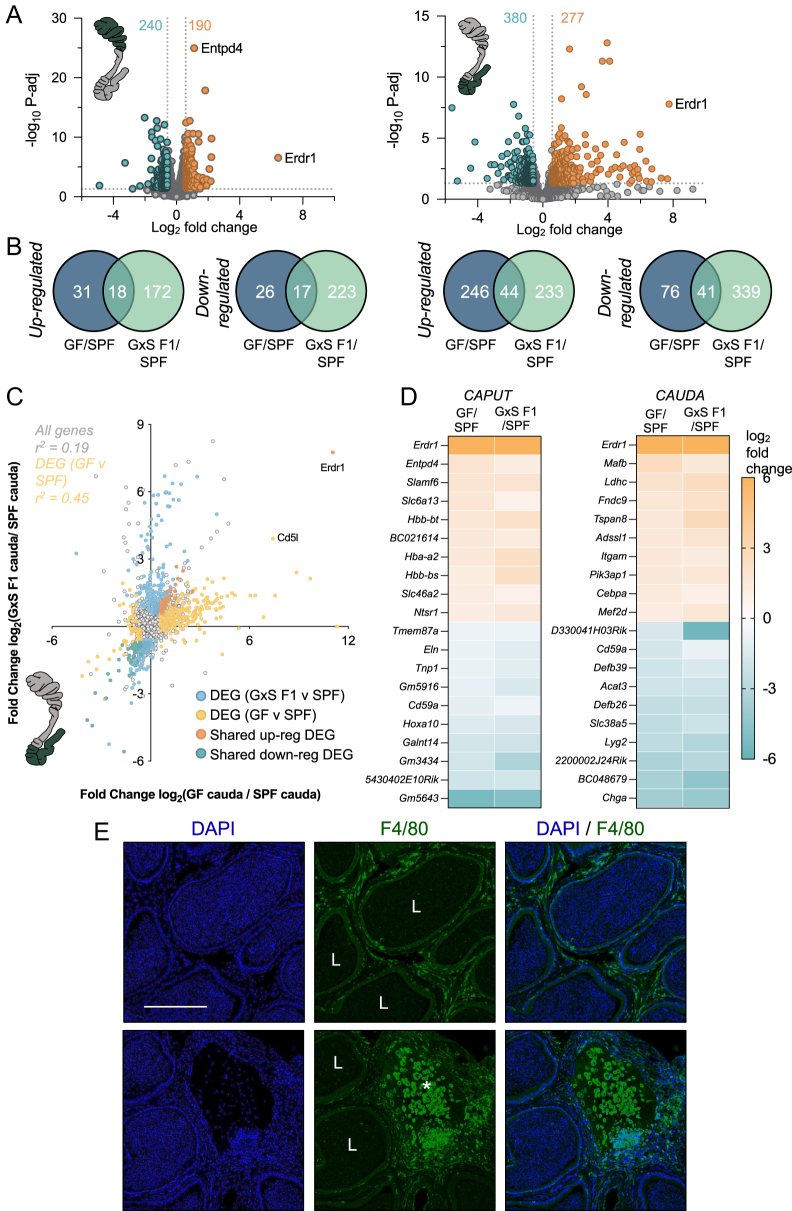
Transcriptomic changes in F1 offspring emulate F0 changes. (A) Volcano plots depicting the log_2_ fold change (*x*-axis) and log_10_ adjusted *P*-value (P-adj; *y*-axis) of genes detected in F1 GF × SPF (GxS) caput (left) and cauda (right) epididymis compared to SPF. Dots highlighted orange and green indicate genes that satisfied the criteria of >±1.5-fold change and *P*-adj ≤0.05 and therefore identified as significantly altered. (B) Venn diagram depicting the number of overlapping DEGs in the caput (left) and cauda (right) epididymis between GF F0 and F1 compared to SPF. (C) Correlation plot of the expression in GF vs SPF (*x*-axis: log_2_ fold change) compared with GxS F1 vs SPF (*y*-axis: log_2_ fold change) cauda epididymis. Blue colored dots indicate GxS F1 vs SPF DEGs, yellow dots indicate GF F0 vs SPF DEGs, while orange and green dots indicate up- and downregulated DEGs shared between GF and GxS F1 offspring when compared to controls. (D) Heatmap of the log_2_ fold change of the top 10 up- and downregulated genes in the caput and cauda epididymis shared between GF and GxS F1 mice. (E) F4/80 (green) and DAPI (blue) immunofluorescent staining in the cauda epididymis of GxS F1 mice. Scale bar = 200 μm. L – lumen and asterisks indicate infiltration of macrophages in the interstitum.

Following the identification of large transcriptomic alterations in the reproductive tract of GF mice compared to SPF males, we next sought to investigate the transcriptomic changes in the epididymis of male offspring produced by a GF male and SPF female breeding pair. Differential analysis revealed 240 downregulated and 190 upregulated genes in the caput epididymis of GF × SPF F1 offspring (Fig. 5A). Of these dysregulated genes, 9.5 and 7% were also significantly altered, respectively, in F0 GF caput compared to SPF mice (Fig. 5B). In line with our differential analysis of F0 tissues, the cauda epididymis of F1 offspring also displayed the greatest number of dysregulated genes. Indeed, overall, 657 genes satisfied our criteria (adjusted *P*-value <0.05, fold change of > ±1.5) of significantly altered. Of these genes, 380 were downregulated and 277 were upregulated in F1 offspring compared to SPF controls (Fig. 5A). Of the genes altered in F0 GF cauda, 15.2 and 34.7% of increased and decreased DEGs, respectively, were also identified to be altered in the cauda of F1 males (Fig. 5B). In extending this comparative analysis beyond the list of DEGs, we compared the fold change of all genes in GF F0 and GF × SPF F1 offspring compared to the SPF caput and cauda epididymis (Fig. 5C and S4D). Strikingly, this revealed a strong correlation of gene alteration between GF F0 and F1 offspring in both regions of the epididymis, with the cauda demonstrating greater correlation. Moreover, analysis of immune cell populations in F1 GF × SPF epididymal tissue revealed a similar trend to GF F0 results (Fig. S4C). Although this result did not reach significance when comparing F1 tissues to controls, we did note altered populations of M2 macrophages trending (*P* = 0.057) in the direction seen in the GF F0 cauda epididymis (Fig. S4E). Furthermore, probing for macrophages (F4/80 immunofluorescence) in histological sections of F1 cauda epididymis revealed a trend of increased abundance of macrophages in the interstitial space (Fig. S4C). Furthermore, we confirmed the presence of cell infiltration in the epididymis of a GF × SPF F1 offspring (Fig. 5E).

As the GF mice used in this study have been maintained in GF conditions for several generations, it is possible that underlying genetic changes in this colony produce the gene expression changes measured in GF and GF F1 male reproductive tissues. However, it should be noted that because GF F1 males are generated by mating GF males with SPF females that any genetic driver of the gene expression changes in progeny would have to result from haploinsufficiency or dominant mutations. Regardless, to rule out the underlying genetic differences in driving the inherited gene expression changes in the male reproductive tracts of GF sired progeny, we performed RNA-seq on tissues collected from recolonized GF mice. These mice were GF mice reared in conventional housing conditions (same as SPF) and breed for over ten generations (Lubin *et al.* 2023). As such, similar gene changes in the male reproductive tract of these mice, compared to our GF and GF F1 males, would suggest a genetic influence rather than a transcriptomic response to the GF environment and non-genetic transmission of altered epididymal gene expression to progeny. While we noted a number of genes, including *Erdr1* and *Entpd4*, similarly altered in the recolonized GF mice, most genes that were persistently altered in F0 and F1 GF mice were not equally affected in the recolonized GF mice (Fig. S3). Thus, highlighting the epigenetically inherited intergenerational impacts of a GF environment.

### A subset of epididymal transcriptomic changes in GF mice are mirrored in mice lacking adaptive immune cells

**Figure 6:**
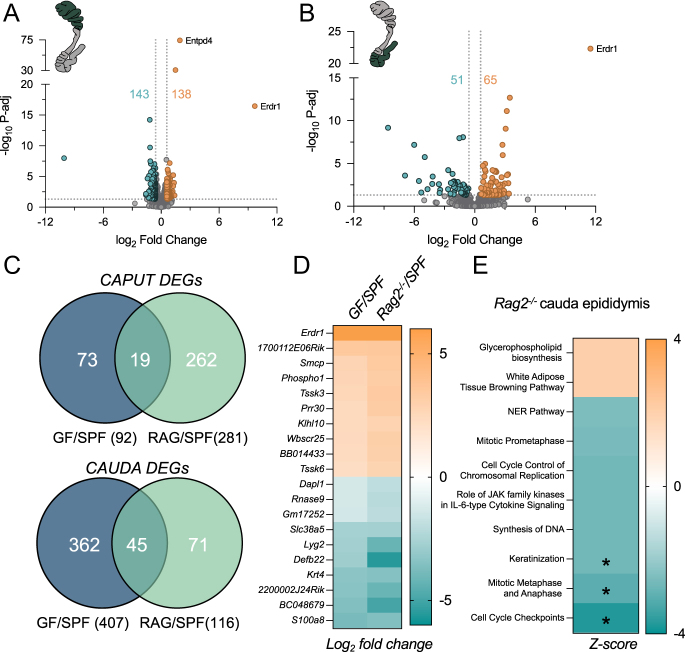
T and B cell-deficient mice display gene expression changes in the epididymis similar to GF. (A, B) Volcano plots depicting the log_2_ fold change (*x*-axis) and log_10_ adjusted *P*-value (P-adj; *y*-axis) of genes detected in the (A) caput and (B) cauda of *Rag2*^−/−^ mice compared to SPF. Dots highlighted orange and green indicate genes that satisfied the criteria of >±1.5 fold change and *P*-adj ≤0.05 and therefore identified as significantly altered. (C) Venn diagram illustrating overlapping DEGs in the caput and cauda epididymis between GF and *Rag2*^−/−^ compared to SPF. (D) Heatmap of the top ten up- and downregulated genes altered in GF and *Rag2*^−/−^ epididymis. (E) Heatmap of the activated (Z-score >2) and inhibited (Z-score <−2) pathways identified by the Ingenuity Pathway Analysis (IPA) software. Asterisks denote pathways equally altered in the GF cauda epididymis.

A recent study identified an epididymal pathology similar to that shown here for GF mice, in mice lacking a subpopulation of T cells with suppressive function known as regulatory T cells (Treg) (Barrachina *et al.* 2023). This study prompted us to consider the role of adaptive immune cells (T and B cells) in driving the dysregulation in the GF epididymis, particularly owing to the well-known association of disrupted T cell function in GF mice (Ostman *et al.* 2006, Harris *et al.* 2024). In support of this, we have also previously demonstrated reduced sebum secretion in *Rag2*^−/−^ mice, which lack T and B cells, akin to GF mice (Choa *et al.* 2021). Moreover, this defective sebum phenotype is also evident in F1 and F2 offspring of *Rag2*^−/−^ males, suggesting a similar non-genetic mechanism of inheritance to that of GF males, leading us to hypothesize a similar impact on the male reproductive tract in *Rag2*^−/−^ mice (Harris *et al.* 2024). Hence, we performed transcriptomic profiling of epididymal tissue from *Rag2*^−/−^ mice and identified a subset of genes that are altered compared to controls (Fig. 6A and B). Of the DEGs identified in the caput and cauda epididymis of *Rag2*^−/−^ mice, 6.8 and 38.8% of these genes were also found to be significantly altered in GF caput and cauda epididymis, respectively (Fig. 6C). Furthermore, we noted the similar increased expression of *Erdr1* and *Entpd4* in *Rag2*^−/−^ epididymis (Fig. 6D). Moreover, of the pathways predicted to be inhibited by IPA in *Rag2*^−/−^ epididymis, three were also identified in the GF cauda epididymis (Fig. 6F, denoted with asterisks). Beyond the pathways shared with the GF cauda epididymis (Fig. 6E, asterisks), DEGs in *Rag2*^−/−^ cauda epididymis were enriched for many additional pathways including glycerophospholipid biosynthesis and cell cycle pathways, such as mitotic prometaphase and synthesis of DNA (Fig. 6E).

## Discussion

The composition and presence of the microbiome plays an important role in human health, including reproductive health. Here, we report that the absence of a microbiome leads to uncontrolled cell infiltration and immune response in the cauda epididymis of GF mice. Moreover, we demonstrate that commensal microbes are not only responsible for influencing the acute changes in the reproductive tract but have a persistent impact on the reproductive tract of the next generation. In further understanding the underlying mechanism, we report similar gene changes occurring in the epididymis of mice lacking T and B cells, implicating the immune system and lymphocyte subtypes in directing epididymal alterations. Ultimately, our results contribute to our understanding of the function of the microbiome in male reproduction, potentially through the regulation of the host immune system.

Despite being long thought to be ‘immune-privileged’, the testis and epididymis are home to a vast array of resident immune cells. Notwithstanding the varying composition along the reproductive tract that directs region-specific functions, these cells collectively are responsible for maintaining tolerance to immunogenic sperm cells and combating incoming pathogens to preserve fertility (Barrachina *et al.* 2023). Thus, in GF mice where immune cell function has been demonstrated to be compromised in somatic tissues such as the intestine and spleen (Macpherson & Harris 2004, Zhang *et al.* 2023), we hypothesized that this dysfunction would extend to the reproductive tract and influence male fertility. Indeed, our results revealed histopathological aberrations in the epididymis and gene expression changes across the tract of GF mice compared to controls. In line with this, a recent paper reported similar phenotypic changes in the epididymis of mice, following selective depletion of a subtype of T cells, regulatory T cells (Treg). Two weeks following Treg depletion, the authors reported a massive influx of macrophages and severe autoimmune epididymitis, a finding strikingly similar to our results in GF males (Fig. 2). Interestingly, the level of Tregs has been previously reported to be reduced in GF lymph nodes by *Foxp3* detection (Ostman *et al.* 2006, Barrachina *et al.* 2023). Furthermore, we demonstrate transcriptomic changes in the epididymis of mice lacking T and B cells (*Rag2*^−/−^ mice) similar to the changes exhibited in the GF epididymis, implicating these immune cell subtypes in directing these gene changes (Fig. 6). Interestingly, the epididymitis-like phenotype was not observed in *Rag2*^−/−^ mice, and while depletion of Tregs led to increased cell infiltration and a pathology not dissimilar to GF mice, the authors reported no damage in the epithelium as evidenced in GF males (Fig. 2). Thus, suggesting the pathology in GF mice that results in the loss of epithelial cell integrity in the cauda epididymis may occur either independent of T cell function or may indicate an advanced stage of the pathology (lack of microbes from birth versus 2-week Treg depletion). Moreover, Treg-deficient mice displayed induced macrophage influx in the testis and caput epididymis, which was not evident in GF mice, suggesting a specific vulnerability of the cauda epididymis to the lack of microbes. This is not the first example of specific sensitivity of the distal region of the epididymis to immunological interventions, with stimuli such as lipopolysaccharide and bacteria also leading to specific inflammatory responses in the distal epididymis, with no visual impact on the proximal epididymis (Silva *et al.* 2018, Wijayarathna *et al.* 2020, Pleuger *et al.* 2022). This region-specific response is potentially explained by a region-specific function and profile of immune cells within the epididymis. Single-cell analysis of immune cells (CD45+) of the epididymis has demonstrated heterogeneity in the population of resident immune cells along the epididymis, suggesting that the unique distribution contributes to different responses seen within each region (Pleuger *et al.* 2022).

Evidence in mice and humans has identified a role for the gut microbiome in male reproduction. Here, we demonstrate reduced testicular weight in mice lacking microbes (Fig. 1C), a finding also evident in mice with gut dysbiosis as a result of antibiotic treatment (Argaw-Denboba *et al.* 2024). Furthermore, comparable to our findings, this study also reported abnormalities in epididymal histology and an altered testicular transcriptome (Argaw-Denboba *et al.* 2024), suggesting that the gut microbiome dysregulation may be the underlying cause of some of the phenotypes seen here in GF mice. We also reported reduced sperm motility in GF mice; however, this did not lead to a concomitant reduction in fertilization with IVF. Albeit sperm motility in GF mice remained at approximately 58%, a score readily associated with successful *in vitro* and *in vivo* fertilization (Sztein *et al.* 2000). Whether the reduced motility impairs fertilization *in vivo* was not thoroughly investigated here; however, the underperformance of GF mice in breeding schemes compared to conventional raised mice has been previously reported. Indeed, GF mice produced an average of 2.5 litters per lifespan compared to 4.7 litters for control mice (Shimizu *et al.* 1998, Munyoki *et al.* 2024). Albeit such schemes included two GF parents and therefore, did not isolate fertility issues associated specific to the paternal microbiome. Ultimately, here, we report the sustained ability of GF male mice to sire offspring and fertilize eggs at similar rates to controls *in vitro*. Conversely, the absence of a microbiome was found to be crucial for maintenance of the ovarian reserve. Indeed, a recent study highlights a reduction in primordial follicle number in GF mice and the role of microbial metabolites, such as short-chain fatty acids in mediating this maintenance (Munyoki *et al.* 2024).

It has become commonly accepted, as a result of hundreds of publications, that the inheritance of phenotypes in the next generation can be non-genetically regulated by the environment experienced by fathers, i.e., a father’s preconception health or lifestyle can influence offspring traits (Soubry 2018). This can manifest in an immediate disease phenotype or susceptibility to disease. For example, mouse models of paternal high-fat diet have demonstrated impaired reproductive function and metabolic disturbances in offspring and grandoffspring (Fullston *et al.* 2012, Fullston *et al.* 2013). Moreover, in a mouse model of early postnatal stress, alterations in behavior and metabolism in offspring persists to the 4th generation in the patriline (Boscardin *et al.* 2022). In some cases, as in the current study, the dysregulation in offspring tissues mimics that of the exposed father, while in others, distinct divergent phenotypes manifest in subsequent generations of progeny than that of the exposed sire. Regarding epigenetic inheritance from paternal microbiome alterations, a recent study utilizing an inducible model of gut microbiota imbalance demonstrated reduced birth weight and increased rate of postnatal mortality in offspring of treated fathers. This increased risk was traced to placental dysfunction in offspring sired by antibiotic-treated fathers and fetal outcomes were ameliorated following gut microbiome restoration (Argaw-Denboba *et al.* 2024). Furthermore, we have previously reported transgenerational epigenetic inheritance from GF fathers, including the transmission of a disrupted sebum secretion phenotype and transcriptomic changes in the liver and spleen of F1, F2 and F3 offspring (Harris *et al.* 2024). Here, we extended the programming of F1 offspring to reproductive tissues and reveal comparable gene expression changes in the epididymis of F1 offspring to that of paternal GF mice, most of which are not altered in recolonized GF mice. Through Ingenuity Pathway Analysis, we identified the altered pathways shared between F0 and F1 GF males to be those involved in cell cycle checkpoints and DNA replication. Pathways implicated in GF cauda epididymis but absent in F1 offspring align with the inflammation and cell influx of GF cauda, which was less evident in F1 cauda, such as phagosome formation and cytokine and macrophage pathways. (Fig. S2F). The persistence of GF programming across multiple tissues of the body has been demonstrated previously by examining the transcriptome of five different GF tissues, including the liver and colon (Mardinoglu *et al.* 2015). Across all five tissues, the authors report two genes that were similarly altered, ectonucleoside triphosphate diphosphohydrolase 4 (*Entpd4)* and nicotinamide nucleotide transhydrogenase (*Nnt)* (Mardinoglu *et al.* 2015). In the current study, we too identified increased expression of *Entpd4,* a gene encoding a protein that catalyzes the hydrolysis of nucleotide diphosphates, in the testis, caput and cauda epididymis (Fig. 4A). This gene was also increased in *Rag2*^−/−^ epididymis and the caput epididymis of GF × SPF F1 mice (Figs 5 and 6). Likewise, erythroid differentiation regulator 1 (*Erdr1*) was equally increased in all GF tissues assayed here (Figs 4A, 5, 6) and previously in liver, skin, intestine and sebaceous glands of GF mice (Harris *et al.* 2024). Moreover, *Erdr1* has been reported to be increased in GF tissues in additional studies (Soto *et al.* 2017). While the significance of these genes being coordinately misregulated in multiple tissues of animals lacking a microbiome or T and B cells is unclear, these genes are the interest of future studies aiming to understand host immune and microbiota interactions that regulate mammalian physiology. Of interest, however, is the equal disruption of *Erdr1* and *Entpd4* in recolonized GF mice (Fig. S3), suggesting a potential genetic impact that persists in GF mice after recolonization in conventional conditions or alternatively that more subtle nuances to the microbiome can influence male reproductive tissues, which requires further investigation.

Ultimately, our findings highlight the role of commensal microbiota and T cells in male reproduction and its non-genetically inherited impact on the epididymis of successive generations. The latter could explain how GF and T and B cell-deficient mice can transmit phenotypes transgenerationally to F2 progeny and beyond (Harris *et al.* 2024). These findings may pave the way into investigating such parameters in other models, including antibiotic treatment or dysbiosis in specific microbiomes (i.e., the gut), to provide translational evidence relevant for human fertility. Nevertheless, our findings support the investigation of the microbiome in a clinical setting as an underappreciated contributor to male reproductive function.

## Supplementary materials











## Declaration of interest

The authors declare that there is no conflict of interest that could be perceived as prejudicing the impartiality of the work reported.

## Funding

NAT is supported by a Lalor Foundationhttps://doi.org/10.13039/100012756 Postdoctoral Fellowship. CCC is the recipient of Pew Biomedical Scholars Award. JCH was supported by the National Institutes of Healthhttps://doi.org/10.13039/100000002 NIAMs F31 fellowship grant (F31AR079845) and T32 training grant (T31AR007465). This work was supported by a Children’s Hospital of Philadelphia Junior Pilot Program awarded to CCC and TK.

## Author contribution statement

Conceptualization was done by NAT, TK and CCC. Methodology was given by NAT, SKZ, TK and CCC. Investigation was done by NAT, SKZ, JCH, MNL and MAN. Formal analysis was done by NAT. NAT, TK and CCC helped in writing the original draft NAT, SKZ, JCH, MNL, MAN, MAS, TK and CCC helped in writing the revision and editing. Supervision of the project was performed by TK and CCC. TK and CCC helped in funding acquisition.

## Data availability

The data discussed in this publication have been deposited in NCBI’s Gene Expression Omnibus and are accessible as of the day of publication using the following accession number GSE269257.

## References

[bib1] Al-Asmakh M, Stukenborg J-B, Reda A, et al. 2014 The gut microbiota and developmental programming of the testis in mice. PLoS One 9 e103809. (10.1371/journal.pone.0103809) 25118984 PMC4132106

[bib2] Ansari N, Müller S, Stelzer EHK, et al. 2013 Chapter 13 – Quantitative 3D cell-based assay performed with cellular spheroids and fluorescence microscopy. Methods Cell Biol etc 113, 295–309. (10.1016/B978-0-12-407239-8.00013-6) 23317907

[bib3] Argaw-Denboba A, Schmidt TSB, Di Giacomo M, et al. 2024 Paternal microbiome perturbations impact offspring fitness. Nature 629 652–659. (10.1038/s41586-024-07336-w) 38693261 PMC11096121

[bib4] Bakos HW, Henshaw RC, Mitchell M, et al. 2011 Paternal body mass index is associated with decreased blastocyst development and reduced live birth rates following assisted reproductive technology. Fertil Steril 95 1700–1704. (10.1016/j.fertnstert.2010.11.044) 21145051

[bib5] Barrachina F, Ottino K, Elizagaray ML, et al. 2023 Regulatory T cells play a crucial role in maintaining sperm tolerance and male fertility. Proc Natl Acad Sci U S A 120 e2306797120. (10.1073/pnas.2306797120) 37676910 PMC10500189

[bib6] Bhushan S, Theas MS, Guazzone VA, et al. 2020 Immune cell subtypes and their function in the testis. Front Immunol 11 583304. (10.3389/fimmu.2020.583304) 33101311 PMC7554629

[bib7] Boscardin C, Manuella F & Mansuy IM 2022 Paternal transmission of behavioural and metabolic traits induced by postnatal stress to the 5th generation in mice. Environ Epigenetics 8 dvac024. (10.1093/eep/dvac024) PMC9730319 36518875

[bib8] Burke ND, Nixon B, Roman SD, et al. 2022 Male infertility and somatic health – insights into lipid damage as a mechanistic link. Nat Rev Urol 19 727–750. (10.1038/s41585-022-00640-y) 36100661

[bib9] Choa R, Tohyama J, Wada S, et al. 2021 Thymic stromal lymphopoietin induces adipose loss through sebum hypersecretion. Science 373 eabd2893. (10.1126/science.abd2893) 34326208 PMC8917823

[bib10] Dimofski P, Meyre D, Dreumont N, et al. 2021 Consequences of paternal nutrition on offspring health and disease. Nutrients 13 2818. (10.3390/nu13082818) 34444978 PMC8400857

[bib11] Fullston T, Palmer NO, Owens JA, et al. 2012 Diet-induced paternal obesity in the absence of diabetes diminishes the reproductive health of two subsequent generations of mice. Hum Reprod 27 1391–1400. (10.1093/humrep/des030) 22357767

[bib12] Fullston T, Teague EMCO, Palmer NO, et al. 2013 Paternal obesity initiates metabolic disturbances in two generations of mice with incomplete penetrance to the F2 generation and alters the transcriptional profile of testis and sperm microRNA content. FASEB J 27 4226–4243. (10.1096/fj.12-224048) 23845863

[bib13] Fullston T, McPherson NO, Owens JA, et al. 2015 Paternal obesity induces metabolic and sperm disturbances in male offspring that are exacerbated by their exposure to an “obesogenic” diet. Physiol Rep 3 e12336. (10.14814/phy2.12336) 25804263 PMC4393169

[bib14] Guiton R, Voisin A, Henry-Berger J, et al. 2019 Of vessels and cells: the spatial organization of the epididymal immune system. Andrology 7 712–718. (10.1111/andr.12637) 31106984

[bib15] Hao Y, Feng Y, Yan X, et al. 2022a Gut microbiota-testis axis: FMT mitigates high-fat diet-diminished male fertility via improving systemic and testicular metabolome. Microbiol Spectr 10 e0002822. (10.1128/spectrum.00028-22) 35446112 PMC9241630

[bib16] Hao Y, Feng Y, Yan X, et al. 2022b Gut microbiota-testis axis: FMT improves systemic and testicular micro-environment to increase semen quality in type 1 diabetes. Mol Med 28 45. (10.1186/s10020-022-00473-w) 35468731 PMC9036783

[bib17] Harris JC, Trigg NA, Goshu B, et al. 2024 The microbiota and T cells non-genetically modulate inherited phenotypes transgenerationally. Cell Rep 43 114029. (10.1016/j.celrep.2024.114029) 38573852 PMC11102039

[bib18] Hou K, Wu Z-X, Chen X-Y, et al. 2022 Microbiota in health and diseases. Signal Transduct Targeted Ther 7 135. (10.1038/s41392-022-00974-4) PMC9034083 35461318

[bib19] Jawaid A, Jehle K-L & Mansuy IM 2021 Impact of parental exposure on offspring health in humans. Trends Genet 37 373–388. (10.1016/j.tig.2020.10.006) 33189388

[bib20] Kennedy EA, King KY & Baldridge MT 2018 Mouse microbiota models: comparing germ-free mice and antibiotics treatment as tools for modifying gut bacteria. Front Physiol 9 1534. (10.3389/fphys.2018.01534) 30429801 PMC6220354

[bib21] Kimmins S, Anderson RA, Barratt CLR, et al. 2024 Frequency, morbidity and equity — the case for increased research on male fertility. Nat Rev Urol 21 102–124. (10.1038/s41585-023-00820-4) 37828407

[bib22] Kostic AD, Gevers D, Siljander H, et al. 2015 The dynamics of the human infant gut microbiome in development and in progression toward type 1 diabetes. Cell Host Microbe 17 260–273. (10.1016/j.chom.2015.01.001) 25662751 PMC4689191

[bib23] Leng L, Metz CN, Fang Y, et al. 2003 MIF signal transduction initiated by binding to CD74. J Exp Med 197 1467–1476. (10.1084/jem.20030286) 12782713 PMC2193907

[bib24] Liang T, Chen J, Xu G, et al. 2021 TYROBP, TLR4 and ITGAM regulated macrophages polarization and immune checkpoints expression in osteosarcoma. Sci Rep 11 19315. (10.1038/s41598-021-98637-x) 34588497 PMC8481262

[bib25] Liu WH, Wang F, Yu XQ, et al. 2020 Damaged male germ cells induce epididymitis in mice. Asian J Androl 22 472–480. (10.4103/aja.aja_116_19) 31696835 PMC7523604

[bib26] Liu BN, Liu XT, Liang ZH, et al. 2021 Gut microbiota in obesity. World J Gastroenterol 27 3837–3850. (10.3748/wjg.v27.i25.3837) 34321848 PMC8291023

[bib27] Love MI, Huber W & Anders S 2014 Moderated estimation of fold change and dispersion for RNA-seq data with DESeq2. Genome Biol 15 550. (10.1186/s13059-014-0550-8) 25516281 PMC4302049

[bib28] Lubin J-B, Green J, Maddux S, et al. 2023 Arresting microbiome development limits immune system maturation and resistance to infection in mice. Cell Host Microbe 31 554–570.e7. (10.1016/j.chom.2023.03.006) 36996818 PMC10935632

[bib29] Luczynski P, McVey Neufeld KA, Oriach CS, et al. 2016 Growing up in a bubble: using germ-free animals to assess the influence of the gut microbiota on brain and behavior. Int J Neuropsychopharmacol 19 pyw020. (10.1093/ijnp/pyw020) 26912607 PMC5006193

[bib30] Macpherson AJ & Harris NL 2004 Interactions between commensal intestinal bacteria and the immune system. Nat Rev Immunol 4 478–485. (10.1038/nri1373) 15173836

[bib31] Mardinoglu A, Shoaie S, Bergentall M, et al. 2015 The gut microbiota modulates host amino acid and glutathione metabolism in mice. Mol Syst Biol 11 834. (10.15252/msb.20156487) 26475342 PMC4631205

[bib32] Miao Y-R, Xia M, Luo M, et al. 2021 ImmuCellAI-mouse: a tool for comprehensive prediction of mouse immune cell abundance and immune microenvironment depiction. Bioinformatics 38 785–791. (10.1093/bioinformatics/btab711) 34636837

[bib33] Montagnoli C, Ruggeri S, Cinelli G, et al. 2021 Anything new about paternal contribution to reproductive outcomes? A review of the evidence. World J Mens Health 39 626–644. (10.5534/wjmh.200147) 33474842 PMC8443996

[bib34] Munyoki SK, Goff JP, Reshke A, et al. 2024 The microbiota extends the reproductive lifespan by safeguarding the ovarian reserve. bioRxiv 2024.2009.2013.612929. 10.1016/j.chom.2025.09.006 PMC13374525 41005310

[bib35] Muscogiuri G, Cantone E, Cassarano S, et al. 2019 Gut microbiota: a new path to treat obesity. Int J Obes Suppl 9 10–19. (10.1038/s41367-019-0011-7) 31391921 PMC6683132

[bib36] Ostman S, Rask C, Wold AE, et al. 2006 Impaired regulatory T cell function in germ-free mice. Eur J Immunol 36 2336–2346. (10.1002/eji.200535244) 16897813

[bib37] Pleuger C, Ai D, Hoppe ML, et al. 2022 The regional distribution of resident immune cells shapes distinct immunological environments along the murine epididymis. Elife 11 e82193. (10.7554/elife.82193) 36515584 PMC9750176

[bib38] Ponnusamy K, Choi JN, Kim J, et al. 2011 Microbial community and metabolomic comparison of irritable bowel syndrome faeces. J Med Microbiol 60 817–827. (10.1099/jmm.0.028126-0) 21330412 PMC3167923

[bib39] Radaelli E, Assenmacher CA, Verrelle J, et al. 2023 Mitochondrial defects caused by PARL deficiency lead to arrested spermatogenesis and ferroptosis. Elife 12 e84710. (10.7554/elife.84710) 37505079 PMC10519710

[bib40] Rahman MS, Pang W-K, Ryu D-Y, et al. 2020 Multigenerational and transgenerational impact of paternal bisphenol A exposure on male fertility in a mouse model. Hum Reprod 35 1740–1752. (10.1093/humrep/deaa139) 32644108

[bib41] Salonia A, Matloob R, Gallina A, et al. 2009 Are infertile men less healthy than fertile men? Results of a prospective case-control survey. Eur Urol 56 1025–1032. (10.1016/j.eururo.2009.03.001) 19297076

[bib42] Sansone A, Di Dato C, de Angelis C, et al. 2018 Smoke, alcohol and drug addiction and male fertility. Reprod Biol Endocrinol 16 3. (10.1186/s12958-018-0320-7) 29334961 PMC5769315

[bib43] Shayeb AG, Harrild K, Mathers E, et al. 2011 An exploration of the association between male body mass index and semen quality. Reprod Biomed Online 23 717–723. (10.1016/j.rbmo.2011.07.018) 22019618

[bib44] Shimizu K, Muranaka Y, Fujimura R, et al. 1998 Normalization of reproductive function in germfree mice following bacterial contamination. Exp Anim 47 151–158. (10.1538/expanim.47.151) 9816490

[bib45] Shin J-H, Park Y-H, Sim M, et al. 2019 Serum level of sex steroid hormone is associated with diversity and profiles of human gut microbiome. Res Microbiol 170 192–201. (10.1016/j.resmic.2019.03.003) 30940469

[bib46] Shiraishi K & Matsuyama H 2018 Effects of medical comorbidity on male infertility and comorbidity treatment on spermatogenesis. Fertil Steril 110 1006–1011.e2. (10.1016/j.fertnstert.2018.07.002) 30396536

[bib47] Silva EJR, Ribeiro CM, Mirim AFM, et al. 2018 Lipopolysaccharide and lipotheicoic acid differentially modulate epididymal cytokine and chemokine profiles and sperm parameters in experimental acute epididymitis. Sci Rep 8 103. (10.1038/s41598-017-17944-4) 29311626 PMC5758752

[bib48] Soto R, Petersen C, Novis CL, et al. 2017 Microbiota promotes systemic T-cell survival through suppression of an apoptotic factor. Proc Natl Acad Sci U S A 114 5497–5502. (10.1073/pnas.1619336114) 28487480 PMC5448176

[bib49] Soubry A 2018 POHaD: why we should study future fathers. Environ Epigenet 4 dvy007. (10.1093/eep/dvy007) 29732171 PMC5920283

[bib50] Sztein JM, Farley JS & Mobraaten LE 2000 In vitro fertilization with cryopreserved inbred mouse Sperm1. Biol Reprod 63 1774–1780. (10.1095/biolreprod63.6.1774) 11090448

[bib51] Ta LDH, Chan JCY, Yap GC, et al. 2020 A compromised developmental trajectory of the infant gut microbiome and metabolome in atopic eczema. Gut Microbes 12 1–22. (10.1080/19490976.2020.1801964) PMC7553750 33023370

[bib52] Tang WH, Kitai T & Hazen SL 2017 Gut microbiota in cardiovascular health and disease. Circ Res 120 1183–1196. (10.1161/circresaha.117.309715) 28360349 PMC5390330

[bib53] Trigg NA, Skerrett-Byrne DA, Xavier MJ, et al. 2021 Acrylamide modulates the mouse epididymal proteome to drive alterations in the sperm small non-coding RNA profile and dysregulate embryo development. Cell Rep 37 109787. (10.1016/j.celrep.2021.109787) 34610313

[bib54] Vijay A & Valdes AM 2022 Role of the gut microbiome in chronic diseases: a narrative review. Eur J Clin Nutr 76 489–501. (10.1038/s41430-021-00991-6) 34584224 PMC8477631

[bib55] Voisin A, Saez F, Drevet JR, et al. 2019 The epididymal immune balance: a key to preserving male fertility. Asian J Androl 21 531–539. (10.4103/aja.aja_11_19) 30924450 PMC6859654

[bib56] Wijayarathna R, Pasalic A, Nicolas N, et al. 2020 Region-specific immune responses to autoimmune epididymitis in the murine reproductive tract. Cell Tissue Res 381 351–360. (10.1007/s00441-020-03215-8) 32383098

[bib57] Yukselen O, Turkyilmaz O, Ozturk AR, et al. 2020 DolphinNext: a distributed data processing platform for high throughput genomics. BMC Genom 21 310. (10.1186/s12864-020-6714-x) PMC7168977 32306927

[bib58] Zatecka E, Bohuslavova R, Valaskova E, et al. 2021 The transgenerational transmission of the paternal type 2 diabetes-induced subfertility phenotype. Front Endocrinol 12 763863. (10.3389/fendo.2021.763863) PMC8602877 34803926

[bib59] Zhang Y, Shen J, Cheng W, et al. 2023 Microbiota-mediated shaping of mouse spleen structure and immune function characterized by scRNA-seq and stereo-seq. J Genet Genomics 50 688–701. (10.1016/j.jgg.2023.04.012) 37156441

