# Supplementary figures and images for: A lack of commensal microbiota influences the male reproductive tract intergenerationally in mice
Source: Reproduction. 2025 Mar 4;169(4):e240204. doi: 10.1530/REP-24-0204 (PMC11906130; doi:10.1530/REP-24-0204)

Supp Figure 1

**A** SPF

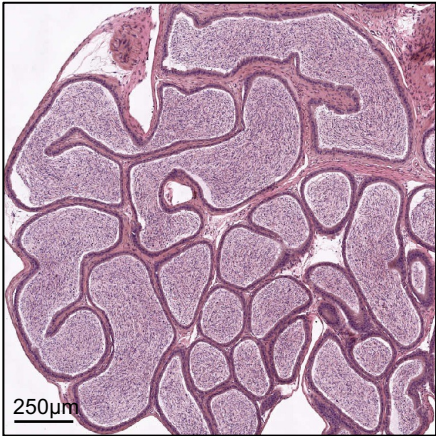

**B** GF

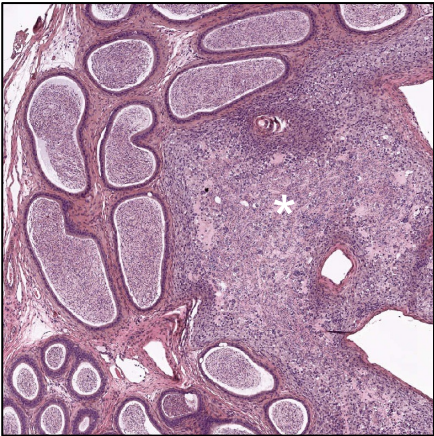

Supplement: Supplementary file 1 [file Supplemental_Figure_1.pdf]

Supp Figure 2

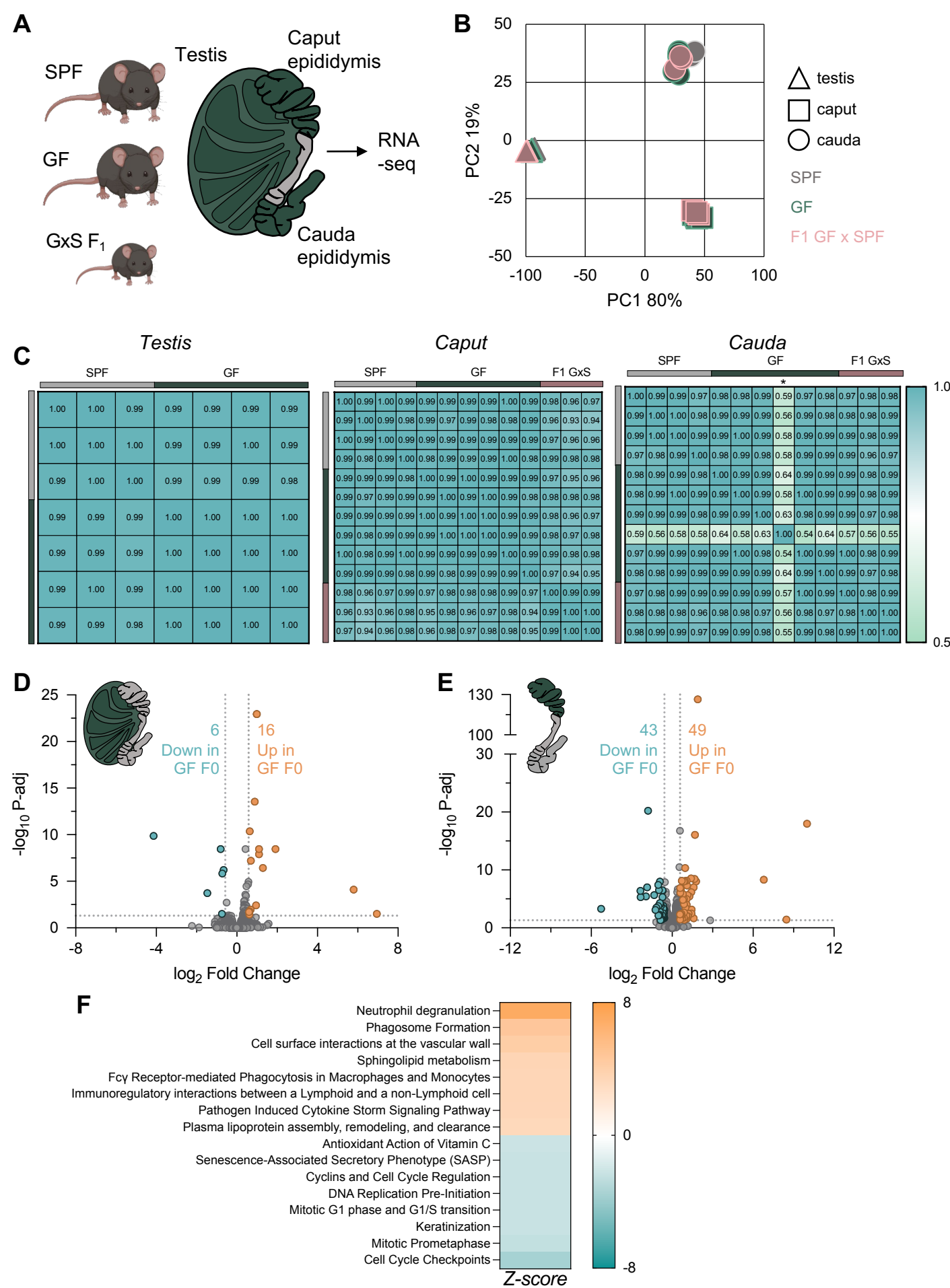

Supplement: Supplementary file 2 [file Supplemental_Figure_2.pdf]

Supp Figure 3

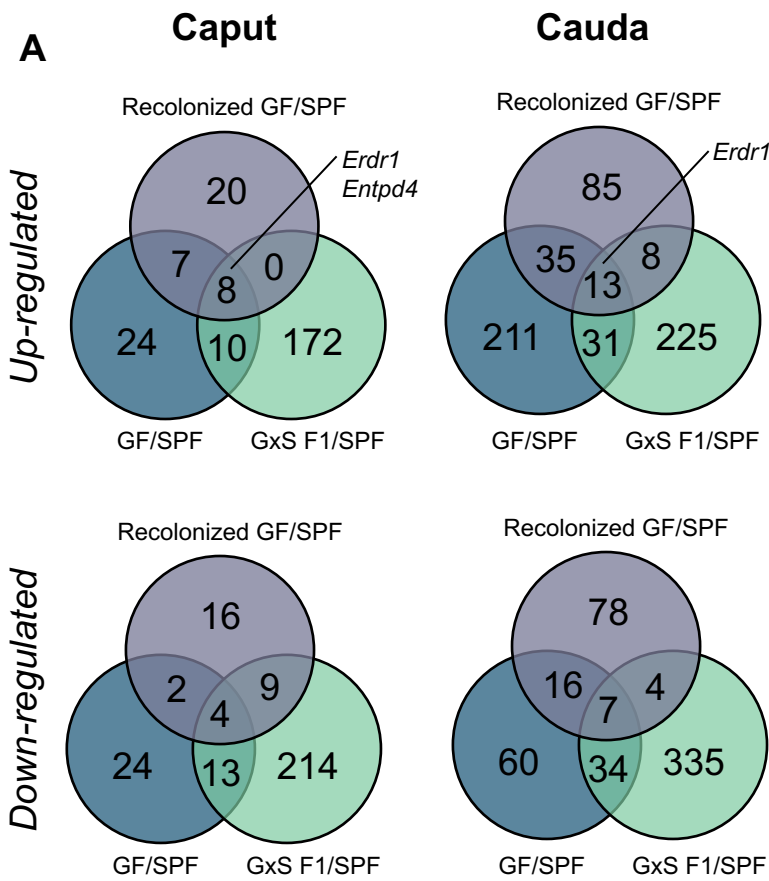

Supplement: Supplementary file 3 [file Supplemental_Figure_3.pdf]

Supp Figure 4

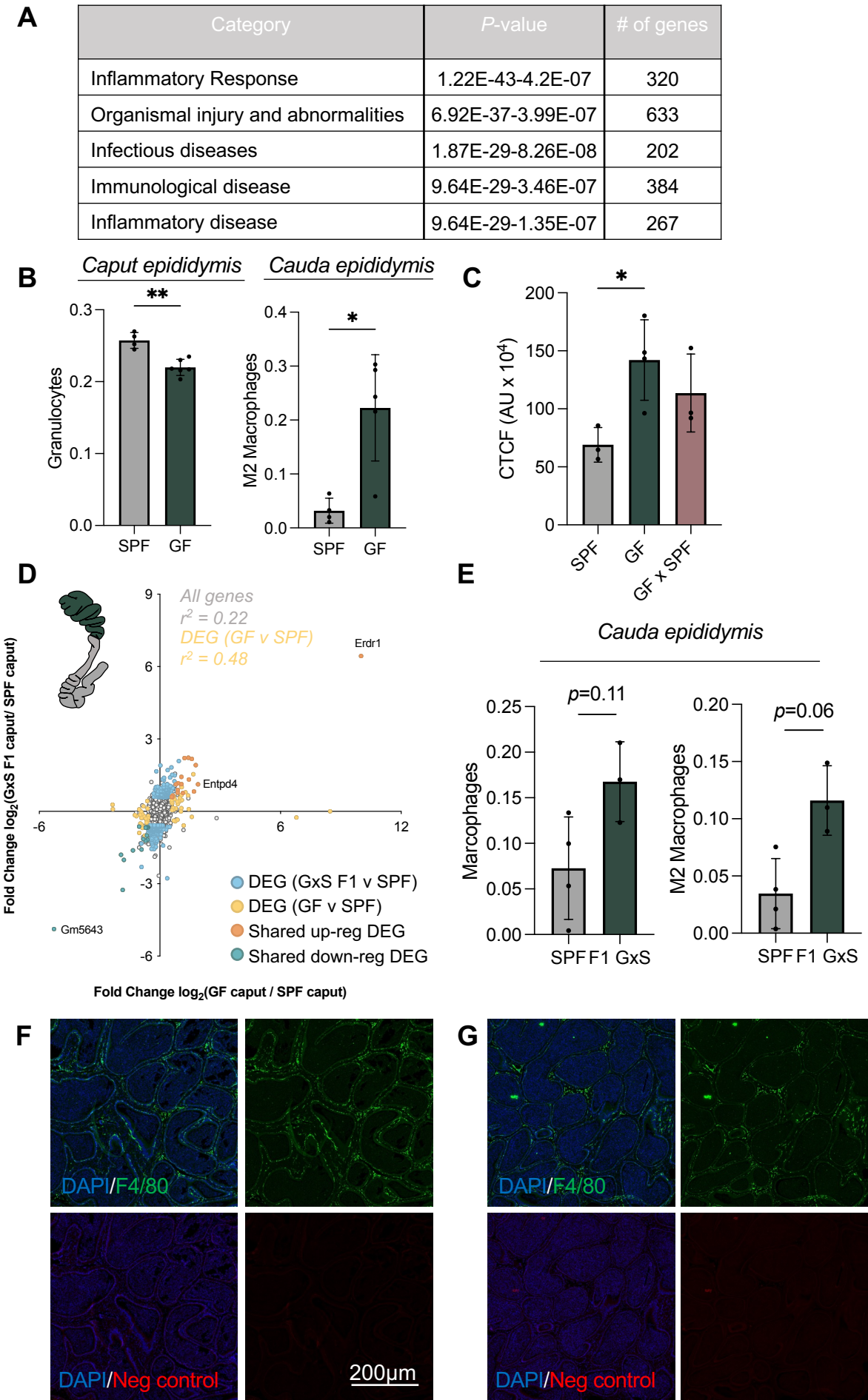

Supplement: Supplementary file 4 [file Supplemental_Figure_4.pdf]
